# Microwave Electrodynamic Study on Antiferroelectric Materials in a Wide Temperature Range

**DOI:** 10.3390/ma15248834

**Published:** 2022-12-10

**Authors:** Pavel Astafev, Aleksey Pavelko, Konstantin Andryushin, Alexander Lerer, Jakov Reizenkind, Larisa Reznichenko

**Affiliations:** 1 Research Institute of Physics, Southern Federal University, Stachki Ave., 194, 344090 Rostov-on-Don, Russia; 2 Department of Physics, Southern Federal University, Zorge Str., 5, 344090 Rostov-on-Don, Russia

**Keywords:** ferroelectric materials, antiferroelectric materials, microwave absorption, PZT

## Abstract

The electrodynamic properties of lead zirconate titanate ceramic solid solutions, exhibiting ferro-antiferroelectric phase transition, are investigated at microwave frequencies in a wide temperature range. Significant changes in the electrodynamic response are found, presumably associated with structural rearrangements accompanying the sequence of phase transitions between para-, ferro-, and antiferroelectric states. The phenomena observed in the experiments are considered under conditions of changing temperature and concentrations of the components; several independent measurement techniques were used for their unambiguous identification.

## 1. Introduction

The main requirements for modern electronic components are miniaturization and reduction of production costs. The use of ferroelectric materials assists in fulfilling these requirements to some extent. In addition, the ability to change the vector of spontaneous polarization or magnetization opens up opportunities for creating devices tunable by an electric field.

Strontium barium titanate is one of the most widely used ferroelectric materials in microwave technology [1]. Its wide application is due to low losses in the microwave range. Nevertheless, attempts are still being made to improve the properties of the basic system, for example, by modifying its structure [2] or complicating the elemental composition and creating composites [3]. Ferroelectric materials based on PbTiO_3_ (PT) and PbZrO_3_ (PZ) can also be used in various electronic components such as resonators, filters, and phase shifters [4,5,6]. For such applications, it is necessary to carry out complex studies of the electrodynamic material’s properties for various compositions in the microwave frequency range using various methods, and it is also necessary to know how the phase and elemental composition, porosity, and other material parameters determined at the manufacturing stage affect its macroscopic properties.

There have already been many studies of such materials in a wide frequency range, for example [7,8]. In addition, much attention is paid to the research of multiferroic materials based on (*x*−1)PbZrO_3_–*x*PbTiO_3_, for example [9,10]. Basically, in such studies, the emphasis is on compositions with outstanding electrical and magnetic characteristics. At the same time, many techniques related to the study of microwave absorption spectra are based on the analysis of the reflection coefficient [11,12], which in the case of materials with a high permittivity, can give incorrect results due to losses during multiple reflections. We are interested in evaluating the applicability of loss analysis methods that take into account both reflection and transmission. We also assume that materials and compositions that do not have outstanding performance at low frequencies may show up at high frequencies, demonstrating unexpected results.

Previously, we studied the microwave absorbing properties of solid solutions of the quaternary system 0.98(*x*PbTiO_3_–*y*PbZrO_3_–*z*PbMg_1/3_Nb_2/3_O_3_)–0.02PbGeO_3_ (PMN-PZT-PG), consisting of ferroelectric (PbTiO_3_), antiferroelectric (PbZrO_3_), and relaxor (PbNb_2/3_Mg_1/3_O_3_–PMN) components [13]. Based on the literature data, it was assumed that these materials can exhibit high microwave absorbing properties. In addition, the presence of a complex structure of the material testified in favor of these assumptions. Besides active components, the material also contained a small amount of germanium oxide, the addition of which improved the processability of the studied solid solutions. The study was conducted using original measurement techniques.

Many compositions with different ratios of components have been investigated; however, the presence of a high level of absorption has not been confirmed.

Despite this, several conclusions were drawn, among which the following can be distinguished:

- Materials with the most diverse phase composition have the best microwave absorbing properties;
- Most of the absorption coefficient maxima are associated with the resonance of electromagnetic waves in the material samples;
- There are correlations between the static permittivity and the position of some absorption coefficient maxima in the frequency domain.

The first conclusion was based on calculating the effective absorption band for all the studied compounds and comparing them with each other. The second conclusion was based on the fact that at the frequency of the minimum of the transmission coefficient, the maximum of the absorption coefficient necessarily occurs (the opposite is not true).

Nevertheless, we still have a number of open questions, for example:

- At which phase transitions (transitions between polar and nonpolar phases, or transitions between different polar phases) does the absorption coefficient increase?
- Which crystal systems present in the morphotropic region have the greatest impact on the radio absorbing properties?
- Is the increasing trend for radio-absorbing properties of the material preserved when it is measured under other conditions (e.g., using a different measuring cell with different reflection and transmission characteristics or a different form of an electromagnetic wave)?

Thus, the purpose of this work was to determine the relationship between the microwave absorbing properties of ferroelectric materials and their microstructure and electrophysical parameters under various external influences. To confirm the conclusions of the previous study and resolve the issues raised, it was decided to conduct a study of a simpler ferroelectric system (*x*−1)PbZrO_3_–*x*PbTiO_3_.

## 2. Materials and Methods

The objects of this study were samples of solid solutions (SS) of the two-component system (*x*−1)PbZrO_3_–*x*PbTiO_3_ (PZT). The materials were obtained by double solid-state synthesis at temperatures of 1140 K and 1170 K, respectively, followed by sintering (temperatures varied in the range (1470–1530) K.) using conventional ceramic technology [14]. X-ray studies and measurements of electrophysical parameters are described in [14,15,16]. The samples were made in the form of cylinders with a diameter of 12 mm and a height of 1 mm. Mechanical treatment of surfaces was carried out with a diamond tool according to the 6th accuracy class. The error of the diameters and thicknesses of the samples was not more than 3% for thickness and not more than 1% for diameter. The number of samples of different compositions utilized in the measurements of microwave absorbing and resonant properties in a wide range of concentrations at room temperature was 98. In this work, the following ranges of PT concentrations were included:

- 0.05 < *x* < 0.30,
- 0.35 < *x* < 0.456,
- 0.495 < *x* < 0.56.

Samples were also measured with the following PT concentrations: 0.01, 0.015, 0.02, 0.6, 0.625, 0.675, 0.725, and 0.8. It was planned to measure the complete system with a step in PT concentration from 0.5 to 1%. However, certain compositions did not survive mechanical processing or measurements at high temperatures. These factors determined the set of samples studied in this work.

In addition, a re-study of the previously studied PMN-PZT-PG system was carried out for the following compositions:

- 0.98(*x*PbTiO_3_–(1−*x)*PbZrO_3_–0.05PbMg_1/3_Nb_2/3_O_3_)–0.02PbGeO_3_, the molar fraction of PT varied in the range (0.37 < *x* < 0.57).
- 0.98(*x*PbTiO_3_–0.05PbZrO_3_–(1−*x)*PbMg_1/3_Nb_2/3_O_3_)–0.02PbGeO_3_, the molar fraction of PT varied in the range (0.11 < *x* < 0.5).
- 0.98(*x*PbTiO_3_–(*x*−1)/2PbZrO_3_–(*x*−1)/2PbMg_1/3_Nb_2/3_O_3_)–0.02PbGeO_3_, the molar fraction of PT varied in the range (0.23 < *x* < 0.52).

When describing the radiophysical studies, the elements of the scattering parameters matrix (S-parameters) are mentioned, so it is necessary to briefly explain their essence. The matrix of S-parameters for a quadripole (two-port device) consists of four coefficients of electromagnetic waves power ratios: *S*_11_ and *S*_22_ are the reflection coefficients for the first and second ports (the power ratio of the reflected wave to the incident wave); and S_12_ and *S*_21_ are the transmission coefficients for the first and second ports (the power ratio of the transmitted wave to the incident wave) [17].

Radiophysical measurements were performed using the vector network analyzer (VNA) P9375A “Keysight” with an operating frequency range of 300 kHz–26.5 GHz. To connect the measuring cell with the VNA measuring cables, microwave adapters of the PC 3.5 standard with a limiting operating frequency of 26.5 GHz were used.

To study the microwave absorbing properties of the material, a measuring cell was used, which is a straight segment of a microstrip line (MSL) on a substrate made of glass fiber reinforced epoxy material (FR4), with coaxial connectors of the SMA 3.5 standard at both ends. The operating frequency range of the connectors is up to 18 GHz. During the measurements, the samples were located in the center of the cell, on the surface of the MSL, and the frequency dependences of the coefficients of the scattering matrix were recorded.

**Figure 1:**
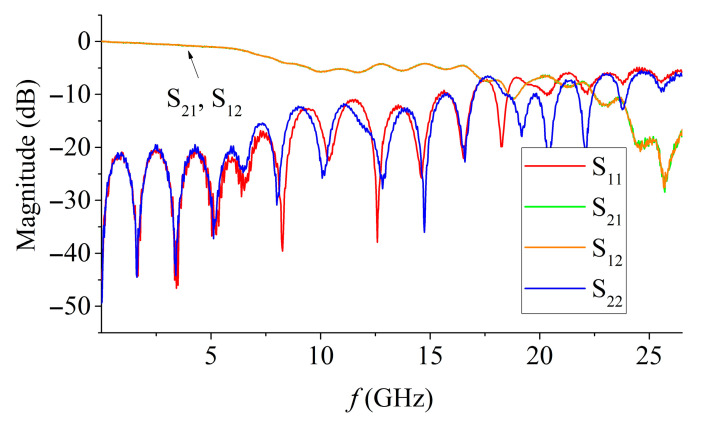
S parameters of MSL.

The contact quality of MSL coaxial connectors and VNA microwave cables, as well as the quality of manufacturing of the measuring cell, can be estimated by analyzing the dependences of the S-parameters on the frequency of an empty measuring cell (Figure 1)

Periodic dips and small ripples of the reflection coefficient are associated with the interference of waves reflected from the coaxial connectors of the MSL and the interference of small reflections from the junctions of the coaxial junctions with the measuring cables and connectors of the measuring cell, respectively. The bandwidth with a satisfactory degree of matching (the level of reflection coefficients does not exceed –20 dB) is about 7 GHz; therefore, the reliability of the measuring the level of the reflection and transmission coefficients is limited from above by a frequency of 7–8 GHz. Although the signal level information may not be accurate, measurements and calculations were still carried out over the entire available frequency range in order to evaluate microwave absorbing and resonant properties at a qualitative level.

The absorption coefficient of the material is defined by the equation:

(1) $$D_{i}=d_{i}-d_{0},$$

where *d_i_* is the scattering coefficient of the MSL with the *i*-th sample, and *d*_0_ is the scattering coefficient of the empty MSL. The scattering coefficients are calculated by the following equation:

(2) $$d=1-{\left|S_{11}\right|}^{2}-{\left|S_{12}\right|}^{2},$$

where *S*_11_ and *S*_12_ are the reflection and transmission coefficient respectively.

The absorption coefficient in our case indicates what part of the energy was dissipated when the sample was introduced into the measuring cell. It consists of three main contributions:

- Energy absorbed by the sample;
- Energy reflected into the surrounding space due to the presence of the sample;
- Energy absorbed by the MSL substrate.

The third contribution may arise due to the fact that the electromagnetic wave reflected by the sample can rush into the MSL substrate.

When analyzing the dependences of S-parameters on frequency, we pay attention to the transmission coefficient, while trying to detect resonant dips. Their presence alone does not provide much useful information; however, if we have two samples of the same shape but different composition in which resonant dips are observed, we can compare some parameters of the samples and materials. In particular, this makes it possible to compare the average value of the permittivity by the dip position in the frequency domain (with a decrease in the permittivity, the minima shift to higher frequencies).

The method for calculating the absorption coefficient makes it possible to compare the level of absorption of electromagnetic waves in the microwave range in various materials with similar electrical parameters. The method for measuring and calculating the absorption coefficient is described in detail in the works [13,18]. In particular, the analysis of the frequency dependences of the absorption coefficient makes it possible to estimate the absorption band width of a given system. By comparing the measurement results of various materials, it can be determined which composition introduces large losses into the system.

**Figure 2:**
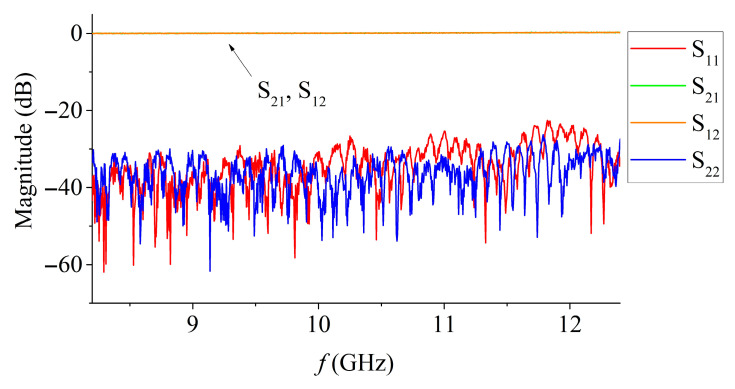
S-parameters of an empty waveguide measuring cell.

To study the microwave absorbing properties of materials as a function of temperature, a measuring cell was used, which is a rectangular waveguide with a cross-section of 23 mm × 10 mm (X-band, 8.2–12.4 GHz) with a heating element isolated by plates of a heat-resistant material. The S-parameters of an empty measuring cell are shown in Figure 2.

In contrast to the method of measuring samples on the MSL, there are no energy losses in the waveguide related to radiation into the surrounding space or losses in the substrate. The first is due to the fact that the electromagnetic wave is concentrated exclusively in the waveguide, the second is due to the absence of a substrate. However, the electromagnetic field is less densely distributed in the waveguide, so it is much more difficult to detect different responses when a sample is introduced into the waveguide.

The main task in measuring samples by this method was to prevent direct contact of the sample with the walls of the waveguide. Touching the walls led to the appearance of non-repeating absorption peaks at different frequencies in the same samples from measurement to measurement. Therefore, in contrast to the method using MSL, in this method, the samples were placed in a rectangular fixture made of heat-resistant material with a hole for the sample inside. The fixture, together with the sample, was located in the waveguide in such a way that the sample was oriented with its faces to the wide walls of the waveguide, and was located in its geometric center.

The calculation of the absorption coefficient was carried out in a similar way as in the case of MSL but additionally took into account the fact that the fixture itself without a sample introduces additional losses in the system, which change as the temperature changes. In the Results and Discussion, we describe this problem in more detail.

## 3. Results and Discussion

Considering the results obtained in the course of the work, the entire study can be conditionally divided into two stages:

- The study of the microwave absorbing and resonant properties of the PZT system in a wide range of concentrations at room temperature;
- The study of the microwave absorbing and resonant properties of 0.97PbZrO_3_–0.03PbTiO_3_ solid solutions in a wide temperature range.

### 3.1. Investigation of Microwave Absorbing and Resonant Properties of the PZT System in a Wide Range of Concentrations at Room Temperature

**Figure 3:**
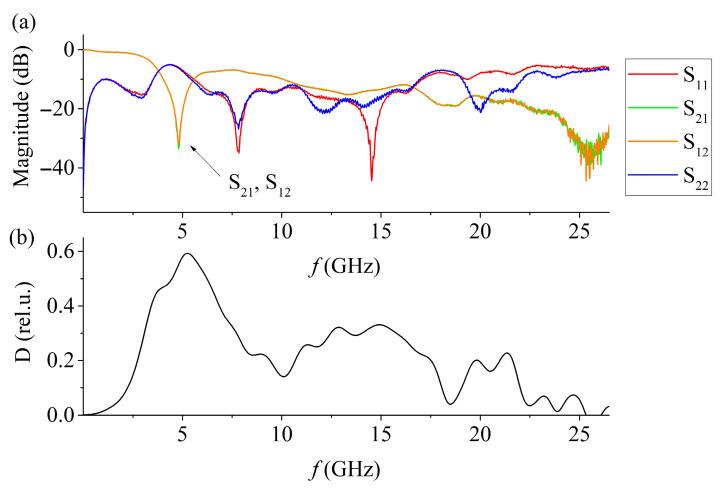
The dependence of the S-parameters (**a**) and the absorption coefficient (**b**) of the MSL with the sample on the frequency.

When the sample is placed on the MSL surface, the response of the entire system changes (Figure 3a). The reflection coefficient minima change position, the overall reflection level increases while the gain level decreases. This is due to the addition of additional reflection points to the system and a change in the phase velocity of the electromagnetic wave inside the sample. In this case, minima of the transmission coefficient appear, presumably associated with the occurrence of electromagnetic resonance inside the cylindrical sample. At frequencies with minimum transmission and reflection coefficients, absorption coefficient maxima are observed (Figure 3b).

The cylindrical shape of the samples was chosen due to the relative ease of their manufacture. Samples of a different shape have similar responses to the microwave electromagnetic field; only the maxima have a different amplitude and are located at different frequencies. Since the methods we use are mainly comparative in nature, a more important issue is the uniformity of the shape of all the samples under study so that they can be compared with each other in many ways.

**Figure 4:**
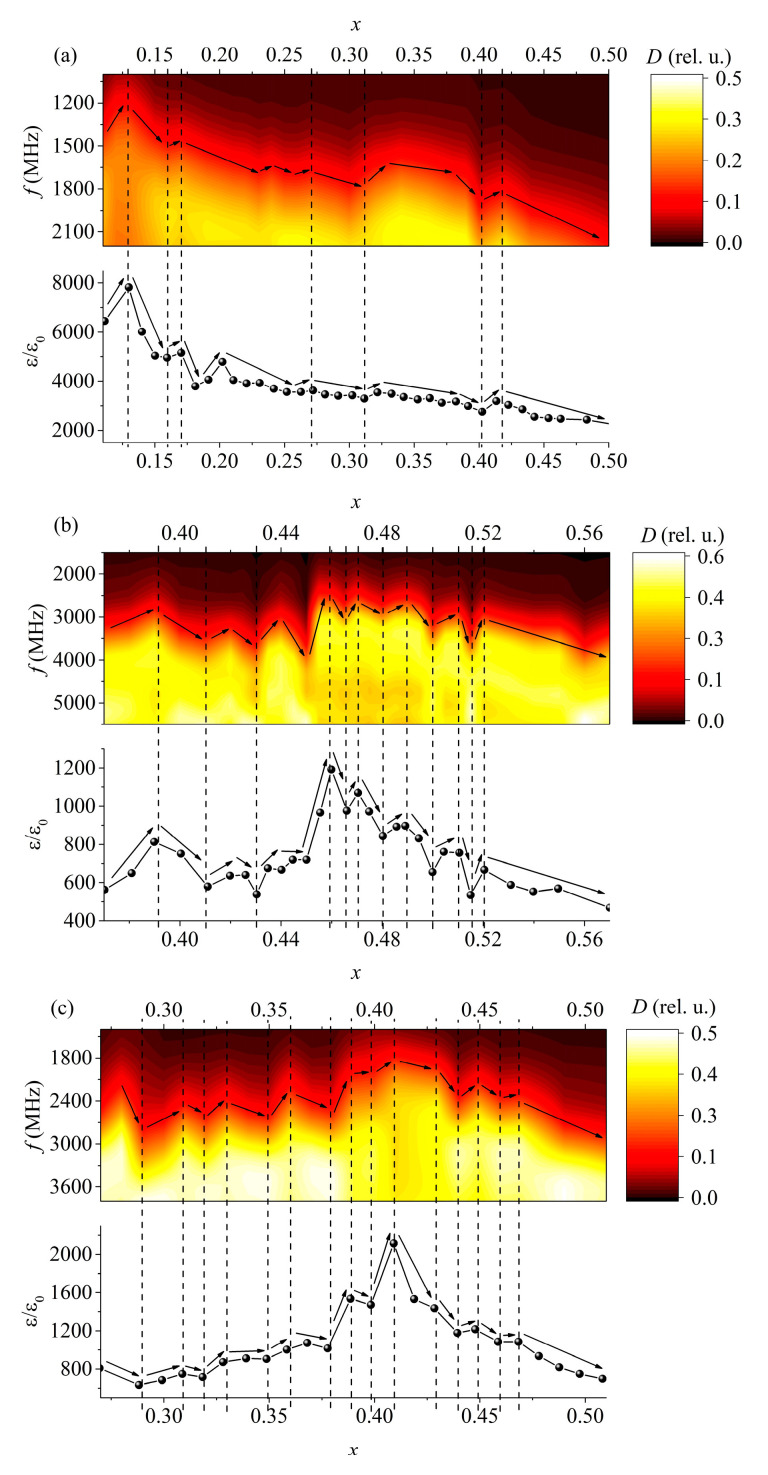
Comparison of the absorption coefficient (*D*) and relative permittivity spectra (*ε/ε*_0_) [19] dependences on the components’ concentration of various PMN-PZT-PG solid solutions: (**a**) 0.98(*x*PT–0.05PZ–(*x* − 1)PMN)–0.02PG; (**b**) 0.98(*x*PT–(*x* − 1)PZ–0.05PMN)–0.02PG; and (**c**) 0.98(*x*PT–(*x* − 1)/2PZ–(*x* − 1)/2PMN)–0.02PG. The dashed line shows the extreme values of the permittivity; the arrows show the trends for the absorption band to shift in frequency and the changes in the permittivity.

**Figure 5:**
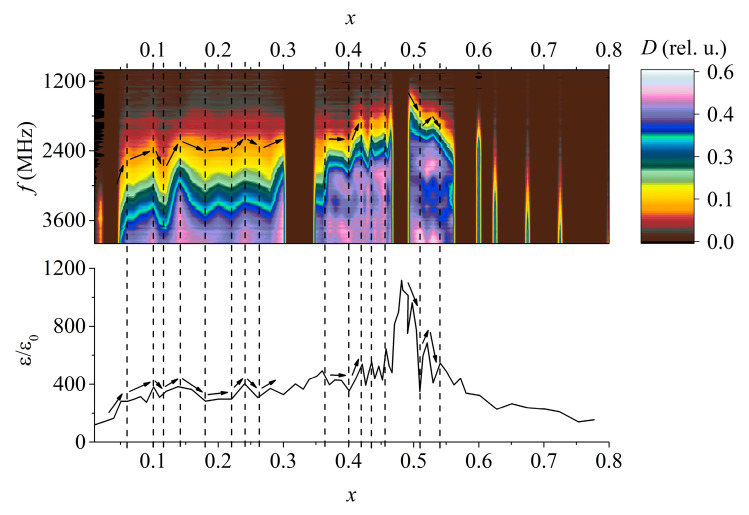
Comparison of the absorption coefficient spectrum (*D*) and the relative permittivity (*ε/ε*_0_) [15] dependences on the concentration of PT in PZT solid solutions. The dashed line shows the extreme values of the permittivity; the arrows show the trends for the absorption band to shift in frequency and changes in the permittivity. For a contrasting display of the absorption band, the drawing is made in a non-standard color scheme. Some SS samples were missing so there are gaps in the absorption coefficient diagram.

Just as in the case of the PMN-PZT-PG system (Figure 4) (the measurements performed in [13] were carried out repeatedly using another device), correlations were also found in this system between the static permittivity and the position of some maxima of the absorption coefficient in the frequency domain (Figure 5). It is worth mentioning that in general, it is incorrect to compare the dependence of the permittivity on the concentration at low frequencies with the dependence of the absorbing properties on the concentration in the microwave range due to the dispersion of the permittivity. However, the fact that the dependence of the position of the maxima in the frequency domain on the concentration almost perfectly coincides with the dependence of the static permittivity on the concentration suggests that the permittivity spectra in certain frequency ranges do not overlap. In other words, if one composition has a greater permittivity than the other at low frequencies, then in the case of the studied ferroelectrics, at high frequencies, of the order of 2–3 GHz, its permittivity is also higher.

**Figure 6:**
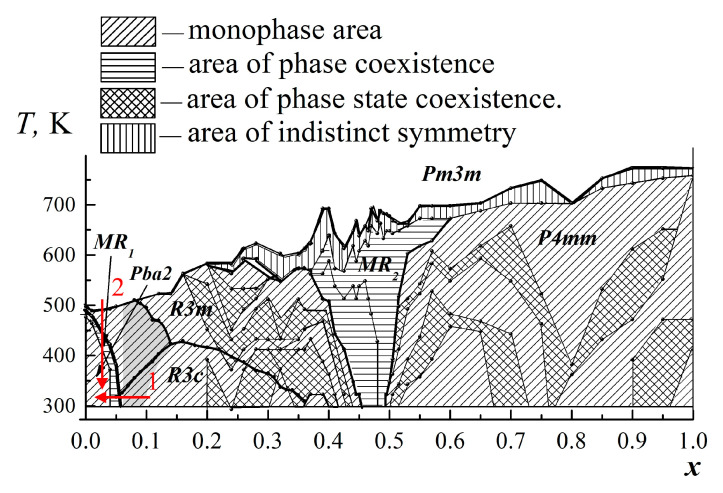
*x*-*T* phase diagram of the PZT system SS samples (Adapted with permission from Ref. [15]. This article was published in “Ceramics International”, 39, Andryushina, I.; Reznichenko, L.; Shilkina, L.; Andryushin, K.; Dudkina, S., The PZT System (PbTi_x_Zr_1 − X_O_3_, 0 ≤ X≤ 1.0): High Temperature X-Ray Diffraction Studies. Complete X-T Phase Diagram of Real Solid Solutions (Part 3), Page No 2889–2901, Copyright Elsevier (2013)). MR1 and MR2 are morphotropic regions. Arrow 1 shows the ferro-antiferroelectric phase transition in concentration; arrow 2 shows the ferro-antiferroelectric phase transition in temperature.

The earlier conclusion that the absorption band width is related to the number of phases in the sample [13] was also confirmed. According to the data presented in [15], the SS of the PZT system at room temperature experiences two phase transitions (Figure 6):

- From the orthorhombic system to the trigonal (rhombohedral) system in the concentration range of PT 0.04 < *x* < 0.06;
- From the trigonal (rhombohedral) system to the tetragonal system in the concentration range of PT 0.45 < *x* < 0.5.

**Figure 7:**
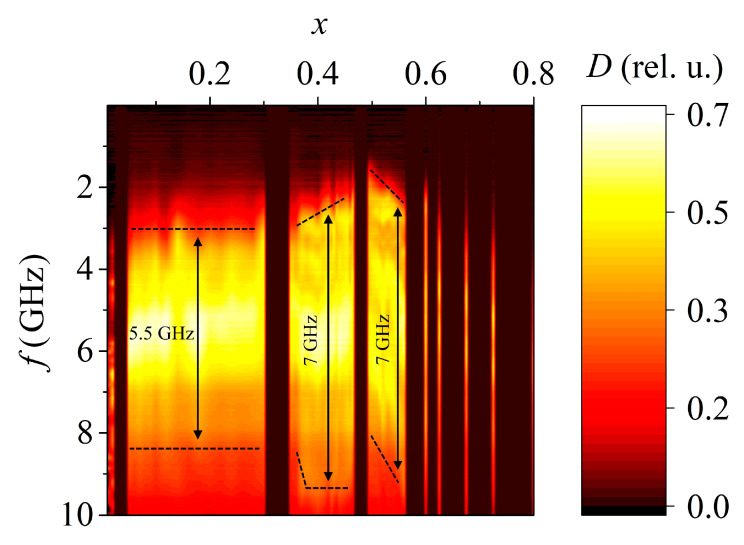
Dependence of the absorption coefficient on the frequency and composition of the PZT system SS samples. The dashed line marks the approximate boundaries of the frequency band with a relatively high level of absorption (more than 40%). Some SS samples were missing so there are gaps in the absorption coefficient diagram.

During these phase transitions, morphotropic regions (MR1 and MR2) appear, in which many different phases coexist, and where an increase in the absorption band width is observed (Figure 7). In our case, only an increase in the absorption band width in the MR2 morphotropic region was confirmed, since in the MR1 region, we had access to samples with too large a step in PT concentration (the MR1 morphotropic region is very narrow).

**Figure 8:**
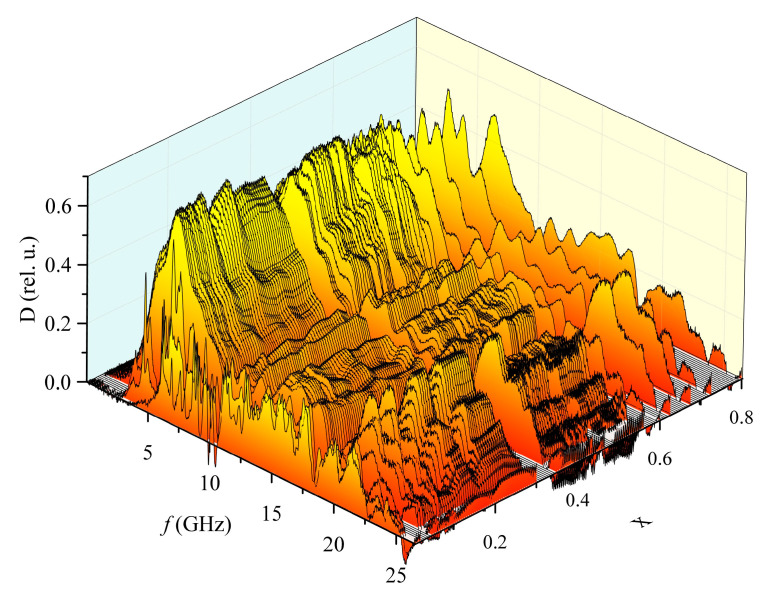
Dependence of the absorption coefficient on the frequency and composition of PZT system SS samples. The color is chosen for contrast, and to make this 3D pattern easier to associate with 2D heat maps.

Upon transition to the antiferroelectric region (*x* < 0.05), the overall level of the absorption coefficient sharply decreases, and the quality factor of the resonant peaks increases (Figure 8).

**Figure 9:**
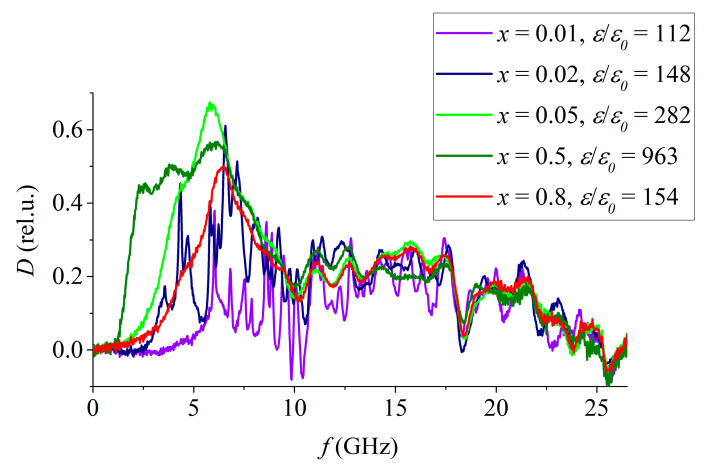
Frequency dependence of the absorption coefficient SS of the PZT system with different concentrations of PT.

These features occur throughout the entire frequency range. They do not correlate with the dependence of the static permittivity on the concentration of PT since samples with concentrations of PT *x* = 0.8 and *x* = 0.02 have approximately the same static permittivity–154 and 148, but their absorption coefficient spectra differ significantly (Figure 9).

**Figure 10:**
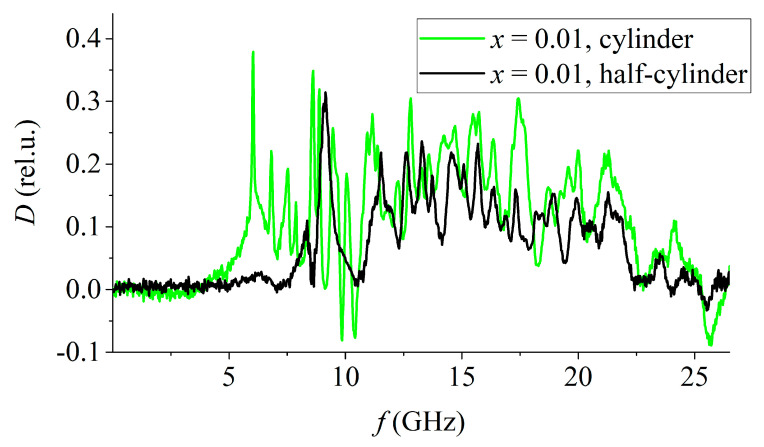
Frequency dependence of the absorption coefficient of SS samples of the PZT system of various shapes.

At the same time, the dependence of the absorption coefficient on the frequency of samples of the composition 0.5PbZrO_3_–0.5PbTiO_3_ has certain similarities with that of samples of the composition 0.2PbZrO_3_–0.8PbTiO_3_, although their static permittivity differs by more than four times. Moreover, samples of a different shape of the same composition (*x* < 0.05) also have high-Q absorption maxima, but at different frequencies (Figure 10). A similar situation is observed for samples of all the remaining compositions: the general form of their absorption spectra remains the same with a change in the shape of the sample, and only the position of the maxima in the frequency domain differs.

Two additional facts need to be taken into account, namely:

- The only difference between the measurements of materials (the different composition of the samples) affects only the phase velocity of the wave in the sample, and changes the nature of the reflection of the electromagnetic wave from the boundaries of the sample;
- All the materials under study do not contain a magnetic subsystem, which suggests that in our case, the permittivity is the only factor affecting the phase velocity and the nature of the reflection,

Therefore, two conclusions can be drawn:

- The relative permittivity of PZT in the antiferroelectric state differs significantly at high frequencies from that in the ferroelectric state, and in the entire studied frequency range, which cannot be said about various ferroelectric phases;
- The occurrence of high-quality resonant maxima depends much more on the material of the sample than on the shape.

### 3.2. Investigation of Microwave Absorbing and Resonance Properties of 0.97PbZrO_3_–0.03PbTiO_3_ Solid Solutions in a WIDE Temperature Range

The transition from the ferroelectric phase to the antiferroelectric phase is also observed in the temperature range (Figure 6). Therefore, a temperature experiment with SS samples of the PZT system with concentrations PT 0.02 < *x* < 0.04 could confirm the fact of a significant change in the resonance pattern upon transition from the ferroelectric phase to the antiferroelectric phase. The use of compositions with a lower or higher concentration of PT at this stage of the study is not advisable since in the first case, the phase transition from the nonpolar phase to the polar one is too close in temperature to the ferroelectric-antiferroelectric phase transition, and in the second, there is a risk of capturing only the morphotropic region and not entering into the antiferroelectric phase at all.

It is not possible to conduct a temperature experiment using MSL because the ferroelectric-antiferroelectric phase transition occurs at temperatures of 400–480 K. Heating MSL to such temperatures will inevitably lead to a change in the characteristics of the substrate or to its destruction. Therefore, it was decided to carry out a temperature study of materials in a section of the waveguide line with a cross-section of 23 × 10 mm (X-band, 8.2–12.4 GHz).

When setting up this experiment, we encountered a number of limitations. The measurements were carried out with a small temperature exposure since a long exposure of the measuring cell to a high temperature can lead to damage to the dielectric inside the coaxial cables near the coaxial-waveguide junctions (heat spreads rapidly from the center of the waveguide to the coaxial cables, while additional cooling of the junctions leads to difficulties in heating the measuring cell). The temperature data were obtained for the outer surface of the waveguide since the creation of a technological hole in the wall of the waveguide to accommodate a thermocouple inside it would lead to additional losses or reflections. Considering that in our case, the fixture already introduces losses into the system, the occurrence of additional sources of losses is highly undesirable. In connection with all of the above, the measurements were taken both during heating and during cooling, in order to evaluate the inertia that could arise due to insufficient time exposure at each temperature point.

**Figure 11:**
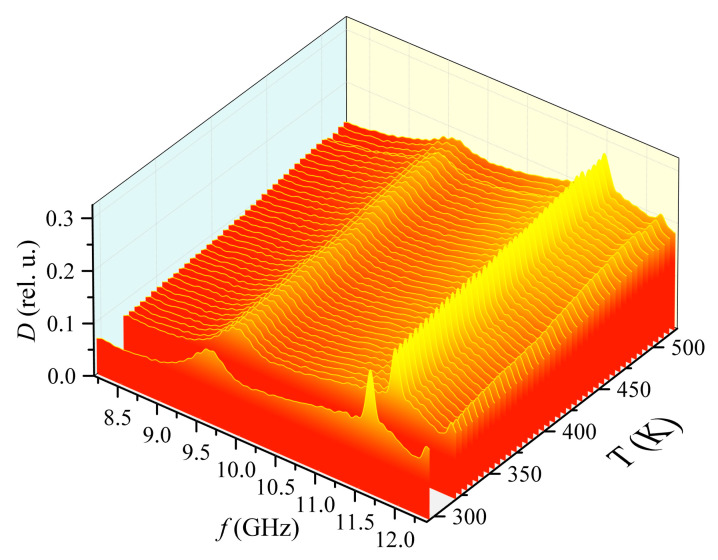
The dependence of the absorption coefficient on the frequency and temperature of the measuring cell with the fixture when heated. The color is chosen for contrast, and to make this 3D pattern easier to associate with 2D heat maps.

Figure 11 shows the dependence of the absorption coefficient of the measuring cell with the fixture without a sample on frequency and temperature during heating. The average level of losses in the system with the fixture is less than 10% and gradually increases with increasing temperature. At frequencies of 9.7, 11.5 and 12.3 GHz, there are absorption maxima associated with the material and shape of the fixture.

**Figure 12:**
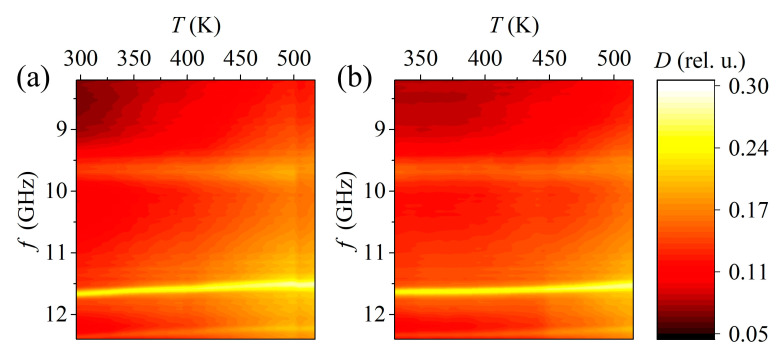
Dependence of the absorption coefficient of the measuring cell with a fixture on frequency and temperature during heating (**a**) and cooling (**b**).

It can be seen from the forward and reverse temperature diagrams (Figure 12) that the maximum at a frequency of 9.7 GHz does not change its position with temperature, but at the same time, the other two maxima gradually shift to the lower frequency region with an increase in temperature. In general, the position of the absorption maxima on the forward and reverse strokes is identical. This indicates there is a slight delay in the increase and decrease in temperature inside the fixture compared with that outside.

An SS sample of the PZT system with a concentration of PT x = 0.03 was chosen as the object for temperature measurements in order to make it possible to carry out the transition from the antiferroelectric phase to the ferroelectric phase in the temperature range of about 400 K during heating.

**Figure 13:**
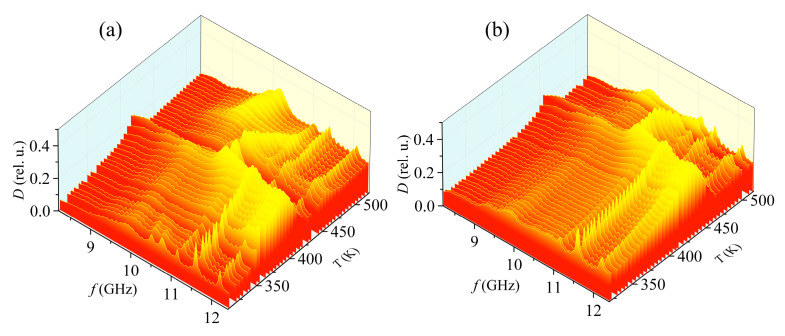
Dependence of absorption coefficient SS of composition 0.97PbZrO_3_–0.03PbTiO_3_ on frequency and temperature during heating (**a**) and cooling (**b**). Three-dimensional diagrams show the general dynamics of changes in the absorption coefficient. The color is chosen for contrast, and to make this 3D pattern easier to associate with 2D heat maps.

Figure 13 shows the dependence of the absorption coefficient of the measuring cell with the SS sample of the composition 0.97PbZrO_3_–0.03PbTiO_3_ on frequency and temperature during heating and cooling. The maxima of the absorption coefficient at frequencies of 11.5 and 12.3 GHz due to the fixture are at approximately the same frequencies. The slight frequency shift is probably due to inaccuracies in the location of the fixture inside the waveguide. In addition to the previously noted maxima, additional maxima appear in the system with the sample at frequencies of 10.5, 10.75, and 11.2 GHz. The maximum at 11.2 GHz practically does not shift in frequency, and its level increases along with the overall level of the absorption coefficient. In this regard, it can be assumed that this maximum is associated with the fixture because its behavior depends on temperature in a way similar to the behavior of other fixture maxima. At the same time, the first two maxima shift to lower frequencies with increasing temperature, while they practically do not change their level and disappear against the background of an increase in the total absorption level. Presumably, these two maxima are of the same nature as the resonances found on the MSL. One way or another, it is impossible to confirm the pattern of occurrence of resonances during the ferro-antiferroelectric phase transition within the framework of this experiment due to the high absorption level of the fixture.

**Figure 14:**
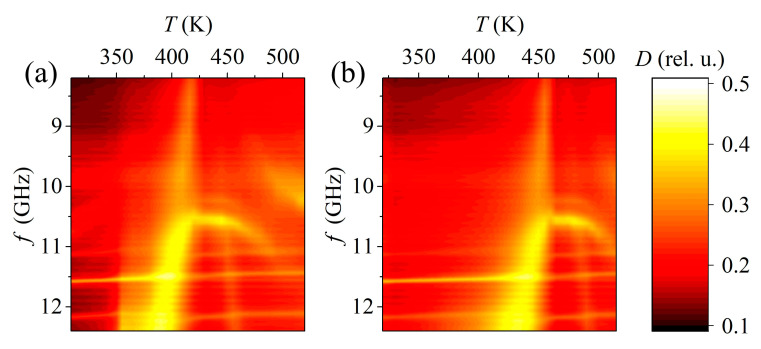
Dependence of absorption coefficient SS of composition 0.97PbZrO_3–_0.03PbTiO_3_ on frequency and temperature during heating (**a**) and cooling (**b**). The thermal maps reflect the positions of all features of the absorption spectrum in the temperature and frequency domains.

When a certain temperature is reached during heating, the absorption level rapidly increases in the high frequency region (Figure 14). With further heating, the frequency band with a high level of absorption expands and shifts to the region of lower frequencies. At a certain point, when heated and cooled, the level of the absorption coefficient drops sharply. Judging by the temperatures at which these sharp changes occur (426 K during heating and 468 K during cooling), these features are associated with the ferro-antiferroelectric phase transition, which is a diffuse first-order phase transition [20]. It is associated with antiparallel displacements of Pb ions and rotations of oxygen octahedra [21]. The authors of [16] note that this transition occurs through the formation of a morphotropic region where an increase in absorption occurs, which once again confirms the fact that the presence of several phases in a material affects its radio absorbing properties.

It is also noted that the driving force of the ferro-antiferroelectric phase transition in PZT is the instabilities of the crystal structure, which manifest themselves through the formation of a sequence of domain transformations. These are most pronounced when a constant electric field is applied, which creates favorable conditions only for some domain configurations [22]. Ultimately, the transition to the antiferroelectric phase occurs from a state characterized by one type of domains. From this point of view, it can be assumed that the increase in absorption in the transition region is associated with energy dissipation on domains or domain walls.

It is also worth noting that, in contrast to MSL studies, in a waveguide line, an increase in the level and absorption bandwidth is observed in the entire frequency range allowed in the waveguide (8.2–12.4 GHz). If we turn to Figure 6, we can see that when measuring SS samples of the PZT system with a concentration of PT 0.4 < *x* < 0.5, which approximately corresponds to MR2, the band with a high absorption level just expands to the high frequency region, up to 10 GHz. Considering the above, it can be assumed that the expansion of the absorption band in the region of the phase transition for materials based on PZT occurs in a fairly wide frequency range up to at least 10–12 GHz. To test this hypothesis, it is necessary to carry out a number of additional experiments in waveguides of a higher and lower frequency range in order to more accurately match the results with measurements on the MSL.

In the ferroelectric region, at a frequency of 10.5 GHz, an absorption maximum appears with a relatively high intensity and a low quality factor, which shifts to higher frequencies and loses intensity as it approaches the nonpolar phase. At a temperature of about 500 K, this maximum disappears. The nature of these maxima is presumably also due to the resonance; however, to reliably confirm this assumption, it is necessary to carry out additional studies using a below-cutoff waveguide. Further heating was also not carried out due to the imperfection of the measuring cell mentioned earlier. Possibly, measurements of samples of other compositions with a higher temperature of the phase transition to the nonpolar region could provide additional information.

## 4. Conclusions

Based on the study of microwave absorbing properties, the following conclusions can be drawn:

- Comparisons of the microwave absorbing properties of samples of different materials on the microstrip line and in the waveguide show that in the morphotropic region, the level and the absorption band width of ferroelectric materials increase significantly. This fact was confirmed for (*x*−1)PbZrO_3_–*x*PbTiO_3_ solid solutions, as well as for related compositions with various modifiers;
- The average absorption level increases in a wide frequency range, as evidenced by a comparison of the experimental results in the waveguide and on the MSL;
- Most of the intense absorption maxima are probably associated with the occurrence of resonance in the samples;
- In solid solutions of the (*x*−1)PbZrO_3_–*x*PbTiO_3_ system and related compositions with various modifiers, which are in the ferroelectric state, there is a correlation between the static permittivity and the position of the absorption band in the frequency range of 1–10 GHz, which indicates that the differences in permittivity at high frequencies are directly proportional to those at low frequencies.

In regards to the study of resonance properties, the following conclusions can be drawn:

- In the PZT compositions, which are in the antiferroelectric state, a significant change in the absorption spectrum was observed in the entire studied frequency range (300 kHz–26.5 GHz), accompanied by a decrease in the total absorption level and an increase in the quality factor of the resonance maxima;
- The ferroelectric-antiferroelectric phase transition led to changes in the resonance pattern of the samples both in the concentration and temperature ranges, which indicates significant differences in the permittivity spectrum of ferroelectrics and antiferroelectrics in the microwave range, in contrast to ferroelectric materials with different microstructures.

As a result, new areas of application of PZT-based materials as resonators or filters in the microwave range could have potential. Moreover, there are suggestions that other antiferroelectric materials can exhibit similar behavior, so it makes sense to consider antiferroelectrics more carefully.

Further refinements within the current experimental framework are proposed:

- The accuracy of temperature measurements can be improved by improving the design of the measuring cell in order to increase the temperature holding capacity. It is also necessary to change the design or material of the fixture to reduce losses. When measuring the absorption coefficient of a material using MSL, the most intense maxima were observed in the frequency range of 3–8 GHz. The use of a waveguide with a wider cross-section for measurements would probably make it possible to confirm the influence of the presence of the antiferroelectric phase on the occurrence of resonances.
- Because an increase in the bandwidth with a high absorption coefficient correlates with the appearance of a morphotropic region, it is possible to conduct a series of temperature experiments with the smallest possible step in concentration, thereby confirming the presence of morphotropic regions and clarifying the position of their boundaries on the diagram. This can only be done with the relative stability of other material parameters, such as relative density, since theoretically, they can also lead to a change in the absorption band width.

Taking into account all of the above, the techniques used in this work can be used as a method for detecting phase transitions in ferroelectric ceramics under certain conditions:

- The unity of the shape of the samples and their position in the measuring cell,
- High accuracy of temperature measurements during the temperature experiment.

However, the high cost of equipment significantly reduces applicability. In our opinion, there are cheaper methods for detecting phase transitions. More important is the fact that these methods make it possible to determine whether there is an abrupt change in the dielectric properties in the microwave range, similar to that observed at low frequencies during phase transitions.

In addition, the use of modeling and new additional research methods can help establish a complete description of the properties of the PZT system, which will make it possible to select the most appropriate compositions for use in certain areas of microwave electronics.

## Data Availability

Not applicable.

## References

[1] Koohi M.Z., Mortazawi A. (2020). Reconfigurable Radios Employing Ferroelectrics: Recent Progress on Reconfigurable RF Acoustic Devices Based on Thin-Film Ferroelectric Barium Strontium Titanate. IEEE Microw. Mag..

[2] Tumarkin A., Tyurnina N., Tyurnina Z., Mukhin N., Sinelshchikova O., Gagarin A., Sviridov S., Drozdovsky A., Sapego E., Mylnikov I. (2020). Barium-Strontium Titanate/Porous Glass Structures for Microwave Applications. Materials.

[3] Repi V.V.R., Manaf A., Soegiono B. (2014). Microwave Properties of Composite Ba_0.5_Sr_0.5_Fe_11._
_7_Mn_0.15_Ti0.15O_19_/La_0.7_Ba_0.3_MnO_3_ Material. Advanced Materials Research.

[4] Nguyen M.D., Tran D.T., Dang H.T., Nguyen C.T., Rijnders G., Vu H.N. (2021). Relaxor-Ferroelectric Films for Dielectric Tunable Applications: Effect of Film Thickness and Applied Electric Field. Materials.

[5] Tagantsev A.K., Sherman V.O., Astafiev K.F., Venkatesh J., Setter N. (2003). Ferroelectric Materials for Microwave Tunable Applications. J. Electroceram..

[6] Özgür Ü., Alivov Y., Morkoç H. (2009). Microwave Ferrites, Part 2: Passive Components and Electrical Tuning. J. Mater. Sci. Mater. Electron..

[7] Macutkevic J., Kamba S., Glemza K., Banys J., Bormanis K., Sternberg A. (2019). High Temperature Dielectric Properties of PMN-PSN-PZN Relaxors. Phys. Status Solidi (b).

[8] Kamba S., Bovtun V., Petzelt J., Rychetsky I., Mizaras R., Brilingas A., Banys J., Grigas J., Kosec M. (2000). Dielectric Dispersion of the Relaxor PLZT Ceramics in the Frequency Range 20 Hz-100 THz. J. Phys. Condens. Matter.

[9] Yao X., Yang Y., Zhang X.-L., Liu Q., Zhou J.-P., Chen X.-M., Zhang G.-B. (2020). Electric and Magnetic Properties of Some Magnetodielectric Composites at Microwave Frequency. J. Magn. Magn. Mater..

[10] Guerra J., McIntosh R., Hernandes A., Guo R., Bhalla A.S. (2015). Frequency Dielectric Response of Ferroelectric–Magnetic Ceramic Composites like PbZr_0.65_Ti_0.35_O_3_–BaFe_12_O_19_. Ceram. Int..

[11] Huang W., Tong Z., Wang R., Liao Z., Bi Y., Chen Y., Ma M., Lyu P., Ma Y. (2020). A Review on Electrospinning Nanofibers in the Field of Microwave Absorption. Ceram. Int..

[12] Wei H., Zhang Z., Hussain G., Zhou L., Li Q., Ostrikov K.K. (2020). Techniques to Enhance Magnetic Permeability in Microwave Absorbing Materials. Appl. Mater. Today.

[13] Astafev P., Pavelko A., Lerer A., Reizenkind J., Noykin Y., Reznichenko L. (2022). Microwave-Absorbing Properties of PbMg_1/3_Nb_2/3_O_3_-PbZrO_3_-PbTiO_3_-PbGeO_3_ (PMN-PZT-PG) Solid Solutions on a Microstrip Line in the Microwave Range. Crystals.

[14] Andryushina I., Reznichenko L., Alyoshin V., Shilkina L., Titov S., Titov V., Andryushin K., Dudkina S. (2013). The PZT System (PbZr_1−X_TixO_3_, 0.0 ≤ X≤ 1.0): Specific Features of Recrystallization Sintering and Microstructures of Solid Solutions (Part 1). Ceram. Int..

[15] Andryushina I., Reznichenko L., Shilkina L., Andryushin K., Dudkina S. (2013). The PZT System (PbTixZr_1−X_O_3_, 0 ≤ X ≤ 1.0): High Temperature X-Ray Diffraction Studies. Complete XT Phase Diagram of Real Solid Solutions (Part 3). Ceram. Int..

[16] Andryushina I., Reznichenko L., Shilkina L., Andryushin K., Yurasov Y.I., Dudkina S. (2013). The PZT System (PbTixZr_1−X_O_3_, 0 ≤ X ≤ 1.0): The Dependences of Electrophysical Properties of Solid Solutions on the Electric Field Strength and Component Concentration (Part 5). Ceram. Int..

[17] Robert E.C. (2001). Foundations for Microwave Engineering.

[18] Reizenkind Y.A., Kleshchenkov A.B., Lerer A.M., Noikin Y.M. A method for comparing active electromagnetic field energy losses in cylindrical samples of active materials placed on top of a microstrip line conductor. Proceedings of the International Symposium: Physics of Lead-Free Piezoactive and Related Materials. Modeling of Eco-Systems (Analysis of Current State and Prospects of Development).

[19] Reznitchenko L., Shilkina L., Razumovskaya O., Yarovtseva E., Dudkina S., Verbenko I., Demchenko O., Yurasov Y.I., Andryushina I., Esis A. (2009). Phase Equilibrium and Properties of Solid Solutions of PbTiO_3_-PbZrO_3_-PbNb_2/3_Mg_1/3_O_3_-PbGeO_3_ System. Inorg. Mater..

[20] Zakharov Y.N., Raevskaya S., Borodin V., Kuznetsov V., Raevskiĭ I.P. (2006). Control of the Temperature Hysteresis and Diffuse Dielectric Anomaly in the Temperature Range of the Ferroelectric-Antiferroelectric Phase Transition in PbZr_1-x_TixO_3_ (0.03 ≤ x ≤ 0.05) Ceramics. Phys. Solid State.

[21] Roleder K., Jankowska-Sumara I., Kugel G., Maglione M., Fontana M., Dec J. (2000). Antiferroelectric and Ferroelectric Phase Transitions of the Displacive and Order-Disorder Type in PbZrO_3_ and PbZr_1−X_Ti_x_O_3_ Single Crystals. Phase Transit. A Multinatl. J..

[22] Vakhrushev S., Andronikova D., Bronwald I., Koroleva E.Y., Chernyshov D., Filimonov A., Udovenko S., Rudskoy A., Ishikawa D., Baron A. (2021). Electric Field Control of Antiferroelectric Domain Pattern. Phys. Rev. B.

